# Vitamin D status in a Brazilian cohort of adolescents and young adults with perinatally acquired human immunodeficiency virus infection

**DOI:** 10.1590/0074-02760150403

**Published:** 2016-02

**Authors:** Annie Schtscherbyna, Carla Gouveia, Maria Fernanda Miguens Castelar Pinheiro, Ronir Raggio Luiz, Maria Lucia Fleiuss Farias, Elizabeth Stankiewicz Machado

**Affiliations:** 1 Universidade Federal do Rio de Janeiro, Hospital Universitário Clementino Fraga Filho, Serviço de Endocrinologia, Rio de Janeiro, RJ, Brasil; 2 Universidade Federal do Rio de Janeiro, Hospital Universitário Clementino Fraga Filho, Serviço de Doenças Infecciosas e Parasitárias, Rio de Janeiro, RJ, Brasil; 3 Laboratório Sérgio Franco, Rio de Janeiro, RJ, Brasil; 4 Universidade Federal do Rio de Janeiro, Instituto de Estudos em Saúde Coletiva, Rio de Janeiro, RJ, Brasil

**Keywords:** adolescent, perinatally acquired HIV, vitamin D, nutrition, Latin America

## Abstract

The purpose was to determine the prevalence and related factors of vitamin D (VitD) insufficiency in adolescents and young adults with perinatally acquired human immunodeficiency virus. A cohort of 65 patients (17.6 ± 2 years) at the Federal University of Rio de Janeiro, Brazil, were examined for pubertal development, nutrition, serum parathormone and serum 25-hydroxyvitamin D [s25(OH)D]. s25(OH)D levels < 30 ng/mL (< 75 nmol/L) were defined as VitD insufficiency. CD4^+^ T-cell counts and viral load, history of worst clinical status, immunologic status as nadir, current immunologic status, and antiretroviral (ART) regimen were also evaluated as risk factors for VitD insufficiency. Mean s25(OH)D was 37.7 ± 13.9 ng/mL and 29.2% had VitD insufficiency. There was no difference between VitD status and gender, age, nutritional status, clinical and immunological classification, and type of ART. Only VitD consumption showed tendency of association with s25(OH)D (p = 0.064). Individuals analysed in summer/autumn season had a higher s25(OH)D compared to the ones analysed in winter/spring (42.6 ± 14.9 vs. 34.0 ± 11.9, p = 0.011). Although, the frequency of VitD insufficiency did not differ statistically between the groups (summer/autumn 17.9% vs. winter/spring 37.8%, p = 0.102), we suggest to monitor s25(OH)D in seropositive adolescents and young adults, especially during winter/spring months, even in sunny regions.

Vitamin D (VitD) regulates bone metabolism and several reports support the hypothesis that this hormone also produces immunomodulatory effects (Holick 2007).

Deficiency of VitD seems to be a major problem worldwide, is highly prevalent in all age groups (Schöttker et al. 2014), and affects both healthy individuals and those with chronic diseases (Weng et al. 2007, Rutstein et al. 2011). Serum 25-hydroxyvitamin D [s25(OH)D] is inversely associated with the frequency of community-acquired pneumonia (Quraishi et al. 2013) and a higher risk of acquisition of other infectious diseases (Watkins et al. 2015). Furthermore, some studies suggest an association between hypovitaminosis D and worst liver fibrosis in patients with chronic hepatitis C infection (Luo et al. 2014).

In human immunodeficiency virus (HIV)-infected women, low VitD levels [s25(OH)D < 32 ng/mL] were associated with an increased risk of vertical transmission of HIV, with faster disease progression and a higher mortality (Mehta et al. 2009, 2010). An American study (Rutstein et al. 2011) showed that 85% or more of children with HIV presented VitD insufficiency [s25(OH)D < 30 ng/mL], which was correlated with moderate or severe immunosuppression. Interestingly, this rate is not different from those reported for healthy adolescents from Europe (González-Gross et al. 2012) or United States of America (USA) (Ginde et al. 2009).

In Brazil, the status of VitD levels in HIV-infected adolescents is unknown. Few studies were performed in healthy adolescents in southeastern states where a tropical climate predominates, showing adequate levels of VitD varying from 28% to less than 10% (Santos et al. 2012, Oliveira et al. 2014). On the other hand, studies with an HIV-infected population were done with adults and women in the Northeast Region of Brazil and low levels of VitD were reported as 24% and 40.7%, respectively (Conrado et al. 2011, Canuto et al. 2015).

Based on all this scientific evidence, the objective of our study was to determine the prevalence of VitD insufficiency and related factors in a sample of adolescents and young patients with perinatally acquired HIV living in a sunny state in Brazil.

## PATIENTS, MATERIALS AND METHODS

*Study population* - All adolescents or young patients (mean age: 17.6 ± 2 years) followed at the Infectious and Parasitic Disease Service of the Clementino Fraga Filho University Hospital of the Federal University of Rio de Janeiro (HUCFF-UFRJ) were invited to participate in this protocol. Exclusion criteria were opportunistic infection, neoplasia (1 case excluded), or current pregnancy. None of the patients enrolled were in use of oral contraceptive, drugs, alcohol, or had other disease that might affect VitD status. One adolescent declared to be a cigarette smoker. None of the patients analysed had ever received bisphosphonates, steroids, VitD, calcium supplements, or any drug that could interfere with mineral metabolism. Blood samples were obtained for VitD analysis from April 2008-May 2011.

Each adolescent ≥ 18 years provided a written informed consent before enrollment. For patients younger than 18 years, written informed consent was obtained from parents/guardians and also the participant’s written informed assent. This research was approved by the Committee of Ethics and Research of the HUCFF-UFRJ (protocol 044/09).

*Data collection and laboratory measurements* - Gender, age, and race (white, mixed race, and black) were collected at the date of the VitD serum collection. However, participants classified as white seem to fit class III of Fitzpatrick classification; mixed race matches classes IV and V, and black would correspond to class VI.

As Rio de Janeiro is a sunny city, with temperatures oscillating between 20ºC in winter and 40°c in summer, people usually wear slight clothes throughout the year. For this reason, the amount of exposed skin was not considered.

Total body mass was measured using a Filizola^®^ platform mechanic scale (Filizola, Brazil) with a maximum capacity of 150 kg (precision of 100 g). Height was obtained using a Tonelli^®^ stadiometer E120A (IN Tonelli, Brazil), 2.20 m in length (precision of 1 mm). All measurements were carried out with participants wearing only slight clothes, barefooted, and no head attire. Measures were done according to standard techniques (Lohman et al. 1988). Body mass index (BMI) was calculated using the anthropometric data. Height-for-age and BMI-for-age Z-scores were compared with the World Health Organization (WHO) charts reference 2007 (Onis et al. 2007). For subjects 19 years of age or older, Z-scores were calculated using the reference values for adolescents 18.9 years of age.

Pubertal development was self-assessed privately using Tanner diagrams (Marshall & Tanner 1969, 1970). Subjects were given a five-stage standardised series of drawings with explanatory texts to assess their own pubertal stage. This method has been previously validated for use in this age group (Morris & Udry 1980).

The dietary assessment was based on a single 24-h recall conducted by a trained registered nutritionist, using measuring cups, spoons, and portion-size images to increase the accuracy of the recall. Analysis of energy and nutrient intake were done using the software Avanutri online (Avanutri Equipamentos de Avaliação, Brazil). Only VitD consumption and its classification according to estimated average requirement are presented here (FNB/IOM 2011). In Brazil there is no nutrient database that analysed VitD in foods. So, in the present study, analysis from this nutrient was done according to food label.

Adolescents also completed the International Physical Activity Questionnaire (IPAQ), a method previously validated for Brazilian adolescents (Matsudo et al. 2001). Patients were questioned about the frequency and duration of activities in the seven days preceding this assessment. Participants were classified in the inactive group (sedentary or insufficiently active), active, or very active (Matsudo et al. 2001).

In the absence of a more reliable method to estimate sun exposure, we chose to use the classification of IPAQ also for this purpose. Our hypothesis was that adolescents with higher physical activity level should be higher in serum VitD because of sun exposure.

Information about antiretroviral (ART) medications was obtained from medical records. Combination ART therapy was considered for those in use of nucleotide reverse transcriptase inhibitors (NRTIs) plus nonnucleoside reverse transcriptase inhibitor (NNRTI), or protease inhibitor (PI). The use of tenofovir (TDF) was considered.

Absolute CD4^+^ T-cell counts, CD4^+^%, quantitative HIV-1 RNA viral load (VL) and log HIV-1 RNA VL were also obtained from medical records, and subjects were categorised based on clinical and immunologic status (CDC 1993). Plasma HIV RNA (copies/mL, cpm) was categorised as undetectable (< 400 copies/mL) vs. detectable. Immunological status using the Centers for Disease Control and Prevention (CDC) criteria for CD4^+^ count was categorised as follows: class 1, no immunosuppression (CD4^+^ > 500 cells/mm^3^); class 2, moderate immunosuppression (CD4^+^ 200-500 cells/mm^3^); class 3: severe immunosuppression (CD4^+^ < 200 cells/mm^3^).

Blood was drawn after overnight fasting for routine exams [calcium, phosphorus, albumin, and parathormone (PTH)] and samples were stored at -80ºC until analysis of hormones [s25(OH)D)]. In this study we just present results from s25(OH)D and PTH.

Serum samples were collected with protection from light and assayed for concentrations of s25(OH)D by high-performance liquid chromatography (Elecsys kit 2010; Roche, Germany) (normal range 30-100 ng/mL). The samples were obtained during any month of the year, but we also evaluated if there was a difference between the VitD status of seropositive individuals when the blood was collected in the summer/autumn season (December-May) with the ones collected in winter/spring (June-November). s25(OH)D levels < 30 ng/mL (< 75 nmol/L) were defined as VitD insufficiency and < 20 ng/mL (< 50 nmol/L) as VitD deficiency (Bischoff-Ferrari et al. 2006). Intact PTH was measured using chemiluminescence (Immulite 2000; Siemens, USA), normal values 12-65 pg/mL.

*Statistical analysis* - The Mann-Whitney *U* or Kruskal Wallis test was employed to verify statistical differences in continuous variables and Fisher’s exact test for categorical variables. Spearman’s coefficients between continuous variables and s25(OH)D were calculated. The level of significance adopted was p < 0.05. Analyses were performed using SPSS v.17.0 (SPSS Inc, Chicago, USA).

## RESULTS

Sixty-five adolescents were analysed. Mean s25(OH)D was 37.7 ± 13.9 ng/mL, 19 (29.2%) of the adolescents and young patients had VitD insufficiency, and among those with insufficiency six (31.6%) had levels compatible with deficiency, one of whom had s25(OH)D < 10 ng/mL.

Six patients presented secondary hyperparathyroidism. We observed a tendency to a negative correlation between s25(OH)D and PTH (r_s_ = -0.282, p = 0.078), that were significant in men (r_s_ = -0.607, p = 0.003). The studied group was categorised according to s25(OH)D level in normal and low VitD (insufficiency + deficiency) (Table). There was no difference between VitD status and gender, age, BMI, nutritional status, clinical and immunological CDC classification, and type of ART. We noticed a higher representation of inactive patients, and higher levels of PTH in the low VitD group, although these differences were not statistically significant. Only VitD consumption showed tendency of association with s25(OH)D (p = 0.064). Spearman linear correlations between 25(OH)D and HIV parameters (CD4^+^ and VL) did not show any correlation.

**TABLE:** Clinical characteristics of Brazilian adolescents and young adults with perinatally acquired human immunodeficiency virus, according to vitamin D (VitD) status (n = 65)

Variables	25(OH)D ≥ 30 ng/mL (n = 46)	25(OH)D < 30 ng/mL (n = 19)	p
Female:male [n (%)]	23 (50):23 (50)	12 (63.2):7 (36.8)	0.416
Age (years) (mean ± SD)	17.6 ± 2.0	17.4 ± 1.9	0.644
Race [n (%)]			0.601
White	18 (78.3)	5 (21.7)	-
Mixed	15 (68.2)	7 (31.8)	-
Black	13 (65)	7 (35)	-
Height-for-age (Z-score) (mean ± SD)	- 1.25 ± 0.81	- 0.81 ± 1.10	0.084
BMI-for-age (Z-score) (mean ± SD)	-0.36 ± 1.12	- 0.47 ± 1.15	0.988
Nutritional status [n (%)]^*a*^			0.796
Severe thinness/thinness	11 (24.4)	4 (21)	-
Normal	27 (60)	13 (68.5)	-
Overweight	7 (15.6)	2 (10.5)	-
VitD consumption (µg) (mean ± SD)	4.5 ± 4.0	2.4 ± 3.0	0.064
VitD consumption adequacy [n (%)]	5 (11.6)	1 (7.7)	1.000
IPAQ [n (%)]			0.112
Very active	8 (18.2)	1 (5.3)	-
Active	28 (63.6)	12 (63.2)	-
Inactive	8 (18.2)	6 (31.5)	-
PTH (pg/mL) (mean ± SD)	34.7 ± 18.6	53.2 ± 34.0	0.145
CD4^+^ (cells/ mm^3^) (mean ± SD)	457.1 ± 287.4	533.2 ± 333.5	0.518
CD4^+^ (%) (mean ± SD)	20.2 ± 10.3	20.6 ± 9.2	0.685
Viral load log^10^ (cpm) (mean ± SD)	1.9 ± 2.0	2.1 ± 1.8	0.832
Undetectable VL [n (%)]^*b*^	20 (46.5)	7 (36.8)	0.583
CDC immunological status [n (%)]^*c*^			0.186
1	1 (2.3)	1 (5.3)	-
2	6 (13.6)	6 (31.6)	-
3	37 (84.1)	12 (63.1)	-
Without ART [n (%)]	2 (4.3)	2 (10.5)	0.574
Use of NNRTIs [n (%)]	7 (15.2)	4 (21.1)	0.718
Use of PI [n (%)]	36 (78.3)	13 (68.4)	0.528
Use of TDF [n (%)]	23 (50)	6 (31.6)	0.273

*a*: nutritional status 1 missing; *b*: viral load (VL) category 3 missing; *c*: immunological status 2 missing; 25(OH)D: serum 25-hydroxyvitamin D; ART: antiretroviral therapy; BMI: body mass index; CDC: Centers for Disease Control and Prevention; IPAQ: International Physical Activity Questionnaire; NNRTIs: nonnucleoside reverse transcriptase inhibitor; PI: protease inhibitor; PTH: parathormone; SD: standard deviation; TDF: tenofovir. p < 0.05.

s25(OH)D was not associated to age, sex, race, nutritional status, or physical activity.

Individuals from the summer/autumn season had a higher s25(OH)D compared to the ones from winter/spring (42.6 ± 14.9 vs. 34.0 ± 11.9, p = 0.011). The frequency of low VitD, despite being less than half of the winter/spring population, did not differ statistically between the groups (summer/autumn 17.9% vs. winter/spring 37.8%, p = 0.102). PTH level did not differ in the different periods, but four patients with secondary hyperparathyroidism collected blood in winter/spring.

## DISCUSSION

In the present study of adolescents and young adults with perinatally acquired HIV we found that 29.2% were VitD insufficiency.

Several epidemiological studies have shown a high prevalence of hypovitaminosis D among healthy adolescents in different regions of the world (Weng et al. 2007, Ginde et al. 2009, González-Gross et al. 2012), including Latin America (Mithal et al. 2009). VitD deficiency has been neglected in the more sunny regions (Maeda et al. 2007). In Brazil there are few studies analysing the status of s25(OH)D in healthy adolescents which makes it difficult to compare our findings. Until now, no study with healthy nonpregnant adolescent or HIV-infected population has been done in the city of Rio de Janeiro. The rates found in our study are lower than the rates reported for other parts of the country. A study with healthy adolescents living in a rural town in the state of São Paulo, Brazil, showed VitD insufficiency in 60% of them (Peters et al. 2009). A study of girls between seven-18 years in the south of Brazil found inadequate levels of s25(OH)D in more than 90% (Santos et al. 2012). Another study in a population of 18-90 years also presented a high level of hypovitaminosis D (77.4%); however, they included a large age range in the study which makes it difficult to compare our results (Unger et al. 2010).

The major source of s25(OH)D is casual exposure to sunlight and the current belief is that this natural source provides adequate serum levels of this hormone for most Brazilians. Rio de Janeiro is located at 22º54’10”S 43º12’27”W, with a tropical climate (varying from 40ºC in summer to 20ºC in winter), and with a considerable sun radiation across the year (Colle & Pereira 1998). Sunlight is abundant even in the winter months and people do expose themselves frequently to the sun, but in the majority of the cases, they study/work indoors. As the duration of the days in summer is longer compared to winter, individuals should have more opportunity to produce VitD in this season. Analysis in the present study suggested that even in Rio de Janeiro there is a difference in s25(OH)D between winter/spring and summer/autumn. These data agree with several other studies, one also in Brazil, and confirm the fundamental importance of the sun’s rays in the formation of VitD stores (Maeda et al. 2007, Unger et al. 2010, Rutstein et al. 2011). Probably, any difference in s25(OH)D between seasons would be justified by the number of sunny days and longer duration of sunlight in summer compared with winter.

In Brazil there is no public policy to enrich food with VitD, so the consumption of this vitamin depends basically on a few food sources like egg yolk, liver, butter, and some types of fatty fish (salmon, tuna, sardines, and mackerel). In some countries, fortified foods specifically labelled as such, including milk and other dairy foods, margarine, and breakfast cereals, are viable options (IOF 2006). The dietary intake of VitD is low in Brazilian adolescents (Peters et al. 2009). The use of VitD supplements is not a common practice, thus, it seems reasonable to assume that low ingestion is one of the causes of our high prevalence of VitD insufficiency in Brazil (Unger et al. 2010) and is contributing to the findings of our study. Previous studies with seropositives children, adolescents, and young adults showed that low dietary intake of VitD is also a common finding in these groups (Stephensen et al. 2006, Arpadi et al. 2009).

Weng et al. (2007) analysing healthy children and adolescent showed in a multivariable model that older age, black race, wintertime, and total daily VitD intake < 200 IU (< 5 µg) were associated with low s25(OH)D concentrations. Different from that in our study, no difference in s25(OH)D was found between white, mixed, and black race, probably because in Brazil there is a high miscegenation.

Few studies have documented the prevalence of VitD insufficiency in adolescents with HIV. According to the REACH study, mean s25(OH)D did not differ significantly between an at-risk for HIV population and HIV+ urban adolescents. The prevalence of VitD insufficiency [s25(OH)D ≤ 15 ng/mL] in this study was 87% (Stephensen et al. 2006). Studies that analysed specifically the HIV population in USA and Canada showed that more than 85% of them presented VitD insufficiency (Kakalia et al. 2011, Rutstein et al. 2011). Another study also in the USA screened 64 children and adolescents (6-16 years) with perinatally acquired HIV living in the city of New York; 8% were found to have severe VitD deficiency [s25(OH)D < 12 ng/mL] and 43% had levels between 12-20 ng/mL. The lower prevalence may be accounted for differences in racial composition of the study participants, time of year of sampling, dietary practices, or amount of sunlight exposure; however, because different assay methods (eg, competitive protein binding, radioimmunoassay) and criteria [s25(OH)D < 15 ng/mL] were used, direct comparisons cannot be made (Arpadi et al. 2009).

Similar to Rutstein et al. (2011), our study did not demonstrate associations between any specific medication or regimen and s25(OH)D status, possibly due to the heterogeneity of treatment regimens and the small sample.

In HIV patients, there are controversies in analysis between immune status and s25(OH)D. Rutstein et al. (2011) found that decreased CD4^+^ count was correlated to s25(OH)D deficiency, but Kim et al. (2012) found no association. Higher s25(OH)D is also associated with lower RNA VL (Kim et al. 2012).

Others factors linked to the HIV virus itself and to the use of ARTs can be considered as additional causes of VitD insufficiency in this specific population. Furthermore, the virus itself decreases VitD levels through the action of proinflammatory cytokines such as tumour necrosis factor*-*α, inhibiting renal hydroxylation, the consumption of s25(OH)D by the macrophages and lymphocytes as the disease progresses, and the type of ART used (Villamor 2006).

PIs block the hydroxylation of s25(OH)D and the bioactivation of 1,25(OH)_2_(1,25 dihydroxyvitamin D) in the kidneys (Cozzolino et al. 2003), while NNRTIs increase the catabolism of s25(OH)D and 1,25 (OH)_2_D (Van Den Bout-Van Den Beukel et al. 2008). Also, VitD is metabolised *via* cytochrome P450, the same pathway as many ART drugs (Welz et al. 2010). TDF, one type of NRTI, may be involved in s25(OH) levels. Higher plasma TDF concentrations were associated with higher VitD binding protein and lower free 1,25-OH(2)D, suggesting a functional VitD deficiency. Different mechanisms that mediate TDF-associated changes in phosphate handling can be enrolled in this association (Welz et al. 2010).

In our study no relation in s25(OH)D and clinical parameters related to HIV (immunological status and ART) were found, in accordance with Chokephaibulkit et al. (2013). Both of these studies enrolled perinatally HIV-infected young patients, so there were no data from naïve patients. The follow-up data of these patients may help understand this lack of association.

In relation to PTH, previous studies have already presented a strong and negative correlation with s25(OH)D, so the PTH level could be used as a marker of s25(OH)D insufficiency (Holick 2007). In our study we also observed a tendency for a negative correlation between s25(OH)D and PTH (r_s_ = -0.282, p = 0.078) in all samples and a significant negative correlation in males (r_s_ = -0.607, p = 0.003).

Our study presents some limitations. We did not quantify the sun exposure, the use of sunscreen, or the air pollution.

Although VitD consumption was the only one parameter analysed that showed tendency of association with s25(OH)D, this result should be carefully extrapolated. This analysis was based in a single 24-h recall, so variations in food consumption may not be revealed.

In conclusion, adolescents and young adults with perinatally acquired HIV in a sunny state had a moderate prevalence of VitD insufficiency. We suggest to monitor s25(OH)D in this population, especially during winter/spring months, even in sunny regions.

Given the global HIV epidemics, and also of low levels of VitD, establishing the clinical significance of optimising VitD status will be of major clinical and public health importance.
